# The sensitivity of the yeast, *Saccharomyces cerevisiae*, to acetic acid is influenced by *DOM34* and *RPL36A*

**DOI:** 10.7717/peerj.4037

**Published:** 2017-11-14

**Authors:** Bahram Samanfar, Kristina Shostak, Houman Moteshareie, Maryam Hajikarimlou, Sarah Shaikho, Katayoun Omidi, Mohsen Hooshyar, Daniel Burnside, Imelda Galván Márquez, Tom Kazmirchuk, Thet Naing, Paula Ludovico, Anna York-Lyon, Kama Szereszewski, Cindy Leung, Jennifer Yixin Jin, Rami Megarbane, Myron L. Smith, Mohan Babu, Martin Holcik, Ashkan Golshani

**Affiliations:** 1 Department of Biology and Ottawa Institute of Systems Biology, Carleton University, Ottawa, Ontario, Canada; 2 Agriculture and Ari-Food Canada, Ottawa Research and Development Centre (ORDC), Ottawa, Ontario, Canada; 3 Children’s Hospital of Eastern Ontario Research Institute, Department of Pediatrics , University of Ottawa, Ottawa, Ontario, Canada; 4 Ottawa Hospital Research Institute, Center for Cancer Therapeutics, Ottawa, Ontario, Canada; 5 Life and Health Sciences Research Institute (ICVS), School of Health Sciences, University of Minho, Portugal; 6 Department of Chemistry, Carleton University, Ottawa, Ontario, Canada; 7 Department of Biochemistry, Research and Innovation Centre, University of Regina, Regina, Saskatchewan, Canada; 8 Department of Health Sciences, Carleton University, Ottawa, Ontario, Canada

**Keywords:** Yeast, Gene deletion, HSP, Protein expression, *Saccharomyces cerevisiae*, Acetic acid, Heat shock, *DOM34* and *RPL36A*, *DOM34*, *RPL36A*

## Abstract

The presence of acetic acid during industrial alcohol fermentation reduces the yield of fermentation by imposing additional stress on the yeast cells. The biology of cellular responses to stress has been a subject of vigorous investigations. Although much has been learned, details of some of these responses remain poorly understood. Members of heat shock chaperone HSP proteins have been linked to acetic acid and heat shock stress responses in yeast. Both acetic acid and heat shock have been identified to trigger different cellular responses including reduction of global protein synthesis and induction of programmed cell death. Yeast *HSC82* and *HSP82* code for two important heat shock proteins that together account for 1–2% of total cellular proteins. Both proteins have been linked to responses to acetic acid and heat shock. In contrast to the overall rate of protein synthesis which is reduced, the expression of *HSC82* and *HSP82* is induced in response to acetic acid stress. In the current study we identified two yeast genes *DOM34* and *RPL36A* that are linked to acetic acid and heat shock sensitivity. We investigated the influence of these genes on the expression of HSP proteins. Our observations suggest that *Dom34* and *RPL36A* influence translation in a CAP-independent manner.

## Introduction

Bacterial contamination is one of the major hurdles behind reduced yield of industrial alcohol fermentation by yeast (Skinner & Leathers, 2004). These infections often compete with ethanol producing yeast for sugars and other nutrients. Certain antibiotics such as virginiamycin are shown to effectively reduce bacterial contamination during alcohol fermentation process (Hynes et al., 1997). However, the use of antibiotics in this manner is not very desirable due to various ecological costs including increased incidence of antibiotic resistance. The use of weak acids such acetic acid has been proposed as an effective alternative to control bacterial growth (Mira, Teixeira & Sá-Correia, 2010).

During industrial fermentation, acetic acid can also be produced as a result of pre-treatment of economical biomass from lignocellulosic plant residues (Klinke, Thomsen & Ahring, 2004). The presence of acetic acid, however, can put a stress on the biology of yeast cells reducing yeast’s fermentation abilities. As an important field of research, the biology of stress has been the subject of vigorous investigations. Although much has been learned over the past decades, many aspects of cellular responses to various stresses remain relatively unknown (Mira, Teixeira & Sá-Correia, 2010; Silva et al., 2013). In general, cells respond to stress in different manners ranging from production of by-products to even programmed cell death (PCD). Previously, the molecular responses to various stressors including acetic acid, heat shock and hydrogen peroxide have been investigated using the budding yeast, *Saccharomyces cerevisiae* as a model system (Silva et al., 2013; Madeo et al., 1999; Ludovico et al., 2001; Ludovico, Madeo & Silva, 2005). Acetic acid has been reported to affect cell viability and trigger PCD. Mechanistically, it has been shown that acetic acid can penetrate into the yeast cells, which leads to intracellular acidification, anion accumulation and inhibition of cellular metabolic pathways (Casal, Cardoso & Leão, 1996).

In eukaryotic systems including mammalian, a number of genes have been implicated in the control of cellular responses to internal and external stimuli through diverse processes (Allam & Ali, 2010; Komar & Hatzoglou, 2011; Thakor & Holcik, 2012). These genes include Hsp90, *c-myc*, Apaf-1, p53, etc., many of which are linked to cell cycle proliferation (Silva et al., 2013; Allam & Ali, 2010; Komar & Hatzoglou, 2011). Hsp90 is a highly abundant and conserved molecular chaperone that plays a central role in a number of cellular processes including cell cycle control, cell survival, signal transduction, intracellular transport, and protein degradation (Jackson, 2013; Shaikho et al., 2016). Hsp90 has two major isoforms: Hsp90*α* which is inducible under stress and Hsp90*β* which is constitutively expressed (Langer, Rosmus & Fasold, 2003; Ahmed & Duncan, 2004). In yeast, there are two Hsp90 homologs, known as Hsc82 and Hsp82, of which Hsp82 is up-regulated in response to the presence of acetic acid and heat shock (Borkovich et al., 1989). In this study, we have identified two yeast genes that are linked to acetic acid and heat shock sensitivity. We further investigated their influence on the expression of Hsp82.

## Materials and Methods

### Yeast strains, media plasmids and primers

Yeast strains are obtained from gene deletion mutant library (haploid deletion set) derived from the *MATa* strain BY4741 (*MATa orfΔ::KanMAX4 his3Δ1 leu2Δ0 met15Δ0*) (Winzeler et al., 1999; Tong et al., 2001) or generated by PCR transformation approach in BY4741 or the *MATα*, BY7092 (*MATα Can1Δ::STE2pr-HIS3 Lyp11Δ leu21Δ0 his31Δ0 met151Δ0*) strains (Tong et al., 2001). YPD, synthetic complete and synthetic drop-out (-ura) media were used as needed. Expression plasmids p281-4-HSP82, p281-4-URE2 and p281 (Silva et al., 2013; Komar et al., 2003) were used for expression studies. pAG25 plasmid was used as a source of nourseothricin (NAT) resistance gene marker in PCR reactions for gene knockout experiments. Plasmids (from *E. coli* and yeast) were extracted using Pure link quick plasmid kit (Invitrogen, Carlsbad, CA, USA) according to the manufacturer’s instruction. The list of primers used/designed in this study is found in File S1.

### Human cell culture and transfection

HeLa cells were acquired from Cedarlane (HeLa ATCC^R^ CCL-2™ ) and were maintained at 37 °C, 5% CO_2_ in complete DMEM media (10% FBS, 1% glutamine, 100,000 U/L penicillin and 100 g/L streptomycin; HyClone). For siRNA knockdown experiments, HeLa cells were seeded at 5 × 10^4^ onto a 6-well plate. The cells were allowed to grow for 24 h at 37 °C before transfection with 10 nM PELO siRNA (cat# sc-91932; Santa Cruz Biotechnology, Santa Cruz, CA, USA) or a non-silencing control siRNA (cat# 102720; Qiagen, Valencia, CA, USA) following the manufacturer’s protocol (Lipofectamine^®^ RNAiMax; Invitrogen, Carlsbad, CA, USA). Cells were harvested 72 h later and analyzed by western blot analysis.

### Yeast gene knockout and DNA transformation

Gene knockout was carried out using LiAc-based method described by Inoue, Nojima & Okayama (1990) and confirmed by colony PCR.

### Quantitative real time PCR (qRT-PCR)

Total RNA was extracted and was converted into cDNA using RNeasy Mini Kit (Qiagen, Valencia, CA, USA) and iscript cDNA synthesis kit (Bio-Rad, Hercules, CA, USA) according to manufacturer’s guidelines. Quantitative PCR was carried out using iQSybergreen master-mix kit (Biorad) according to the manufacturer’s instruction on a Rotor Gene 3000 (Corbett Research). Thermo cycler conditions were set to the following: 50 °C for 2 min, 95 °C for 10 min, 40 cycles of 95 °C for 30 s-60 °C for 30 s-2 °C for 30 s and a final 72 °C for 10 min (Pfaffl et al., 2001; Yu et al., 2007). PGK1 was used as a housekeeping gene in qRT-PCR experiments (Chambers et al., 1989; Samanfar et al., 2013; Samanfar et al., 2014).

### *β*-*galactosidase* assay

*β*-*galactosidase* assay was performed using ONPG (O-nitrophenyl-beta-D- galactopyranoside) as a substrate as explained in Lucchini et al. (1984) and Stansfield, Akhmaloka & Tuite (1995). When required, cells were exposed to 2 h acetic acid (220 mM) before induction by galactose.

### Drug sensitivity analysis (Spot test)

Yeast strains were grown to the mid-log phase. For acetic acid sensitivity they were challenged for 2 h in YPD liquid media containing 220 mM acetic acid and serially diluted (10^−2^–10^−5^). A total of 15  μl of each dilution was plated on solid media and incubated in identical conditions at 30 °C for 2 days (in triplicates) as described by Silva et al. (2013). For heat shock analysis, cells were challenged for 2 h at 45 °C, serially diluted as above and incubated at 37 °C for 2 days (in triplicates). For control conditions, acetic acid and heat shock treatments were omitted and the plated cells were incubated at 30 °C for two days. For different growth conditions (treatment versus control), the size and number of colonies formed under different cell dilutions were used as a measure to evaluate strain sensitivity. -ura selective drop-out liquid media were used for the overnight growth of yeast strains that carried expression plasmids.

### Western blot analysis

HeLa cells were washed with PBS, scraped, and transferred to an Eppendorf tube. Cells were pelleted and resuspended in RIPA buffer (50 mM Tris–HCl [pH 7.4], 1 mM EDTA, 150 mM NaCl, 1% NP-40, 0.5% SDS, 1 mM PMSF) for 30 min on ice. Lysates were centrifuged at 12,000 × g for 15 min to pellet cell debris. Bichoninic acid assay (BCA, Thermofisher) was used to quantify protein concentration and equal concentrations were loaded on 10% SDS-PAGE gels. Proteins were transferred to a PVDF membrane and analyzed with the following antibodies: mouse anti-HSP90 (CAT# 386040; Calbiochem, San Diego, CA, USA), mouse anti-PELO (CATt# sc-393418, Santa Cruz Biotechnology, Santa Cruz, CA, USA), and mouse anti-*β*-Actin (Abcam, CAT# ab6276), followed by anti-mouse HRP-conjugated secondary antibodies (Cell Signaling Technology, Danvers, MA, USA). Antibody complexes were detected using an ECL (GE Biosciences) and exposure to film. For quantification purposes Alexa 680- or Alexa 780-conjugated (LI-COR Biosciences, Lincoln, NE, USA) secondary antibodies were used followed by detection using LI-COR Odyssey infrared scanner (LI-COR Biosciences). Densitometry analyses were accomplished using the LI-COR Odyssey software.

### Genetic interaction analysis

Synthetic genetic array (SGA) analysis was performed and analyzed as describe by Tong et al. (2001), Samanfar et al. (2013) and Samanfar et al. (2014). In brief, the query genes *DOM34* and *RPL36A* were replaced with the nourseothricin-resistance (NAT) marker in the haploid *MATα* strain, BY7092. The generated gene deletion strains were crossed to two arrays of gene knockout strains of haploid *MATa* mating type. One of these arrays, termed the translation array, contained 384 deletion strains for genes that were directly or indirectly linked to the process of translation. The second array, termed the random array, contained 384 randomly selected gene deletion mutants and was used as a control. Each mutant strain in the translation and random arrays carries a kanMX resistance marker used to replace a target gene. After a few rounds of selection, haploid strains of a-mating type that carry both gene deletions were selected. Colony size measurement was used as a measure of fitness for each strain as in Memarian et al. (2007). The experiment was repeated three times and those interactions that showed growth reduction of 20% or more in at least two experiments were considered hits and were subjected to confirmation using random spore analysis. To improve coverage, we combined our interaction data with those previously reported (http://drygin.ccbr.utoronto.ca). Conditional SGA was performed in the presence of a mild sub-inhibitory targeted condition as in Kumar et al. (2016). For this purpose, 110 mM acetic acid, 20 ng/ml cycloheximide, 10 mg/ml paromomycin and 2 ng/ml rapamycin were used. For heat shock condition plates were incubated at 34 °C. PSA (Phenotypic Suppression Array) analysis was performed as described by Sopko et al. (2006), Alamgir et al. (2008) and Samanfar et al. (2014) in the presence of a strong sub-inhibitory targeted condition. Each experiment was repeated three times. Deletion mutant strains with 20% or more improved fitness in at least two experiments were considered hits and were subjected to confirmation using spot test analysis. The phenotypic suppression conditions were as follows: acetic acid (220 mM), cycloheximide (60 ng/ml), paromomycin (22 mg/ml), rapamycin (6 ng/ml) and heat shock (37 °C).

### Statistical analysis

One-way ANOVA was used to evaluate differences between mean values of different experiments. All experimental results were obtained through a minimum of three independent repeats.

## Results and Discussion

### Deletion of *DOM34* or *RPL36A* increases yeast sensitivity to acetic acid

Acetic acid treatment is proposed as a potential method to control bacterial growth during industrial alcohol fermentation by yeast. Pre-treatment of economical biomass from lignocellulosic plant residues also results in high levels of acetic acid by-products (Klinke, Thomsen & Ahring, 2004). The presence of acetic acid, however, can put a stress on yeast cells. When cells are treated with acetic acid, general translation shuts down (Almeida et al., 2009). In contrast, however, expressions of both *HSC82* and *HSP82* heat shock genes are up-regulated. Deletion of *HSC82* or *HSP82* alters sensitivity to acetic acid and heat shock treatments (Silva et al., 2013). *HSC82* and *HSP82* arose from yeast whole genome duplication and code for paralog cytoplasmic Hsp90 family of proteins. They share 97% sequence identity and together the encoded proteins compromise 1–2% of total yeast proteins. *HSC82* is expressed constitutively at high levels and is slightly induced by heat and stress whereas *HSP82* is strongly induced by heat and stress. *HSC82* and *HSP82* are required for the activation of a number of key cellular regulatory proteins like transcription factors and kinases including Hap1 zinc finger transcription factor involved in regulation of gene expression in response to levels of heme and oxygen and Swe1 protein kinase that regulates G2/M transition (Burnie et al., 2006).

**Figure 1:**
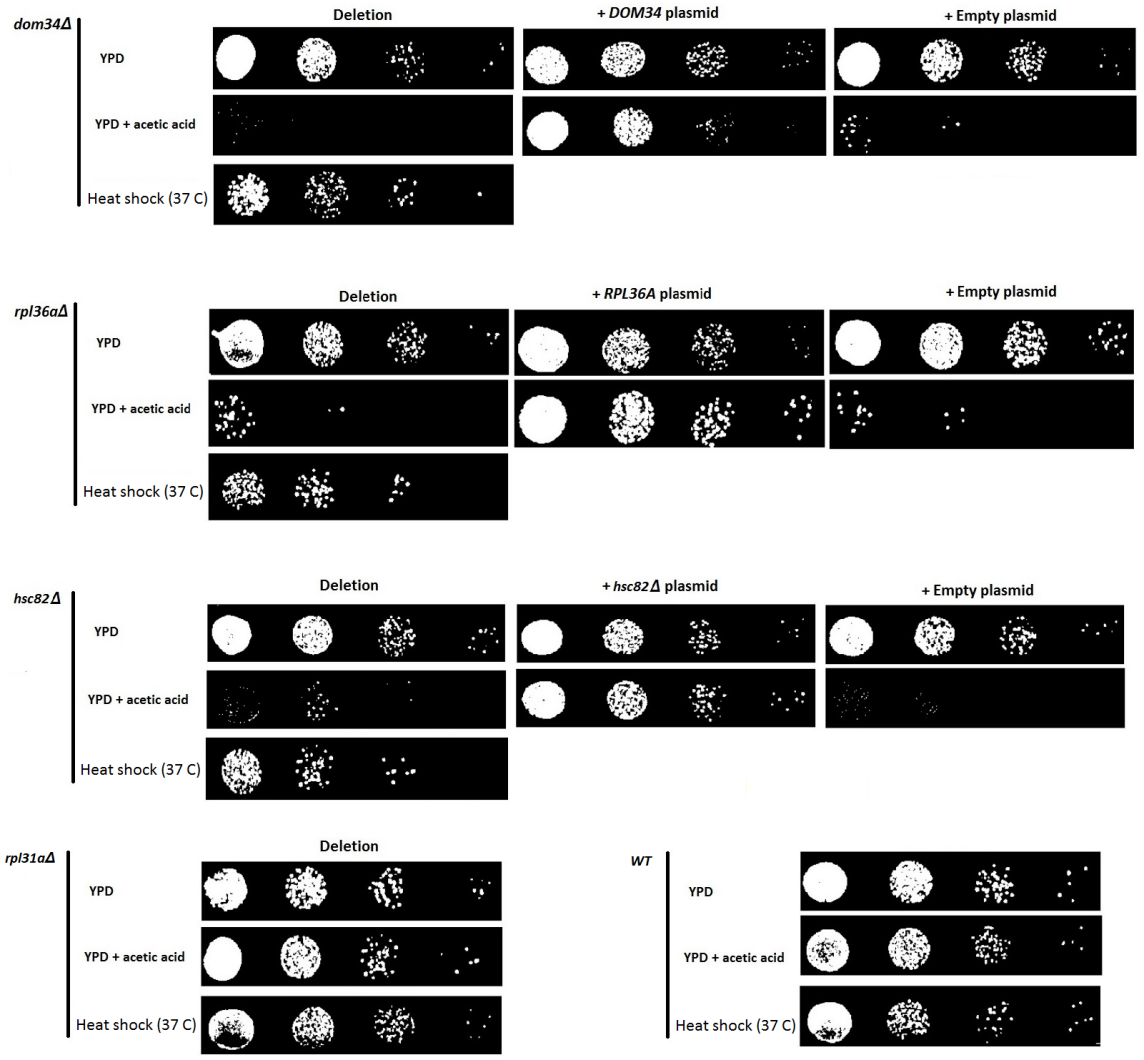
Evaluating the sensitivity of different strains to acetic acid and heat shock treatments. Deletion of *DOM34* and *RPL36A* results in increased sensitivity to acetic acid and heat shock treatments. Reintroduction of the deleted genes converted the sensitive phenotypes to the *WT* sensitivity level. All sensitivity analyses are performed in triplicate with similar results. Acetic acid treatment at concentration of 220 mM was used for two hours. Heat shock was performed at 37 °C. Deletion of *RPL31a* is used as a representative negative control to indicate that acetic acid sensitivity is not correlated with defective translation.

Recently, it was shown that the up-regulation of *HSC82* and *HSP82* in response to acetic acid exposure is controlled at the translation level in a mRNA 5′ CAP-independent manner representing a compelling mode of gene expression control (Silva et al., 2013). Using this mode of gene expression control, it appears that yeast can up-regulate the expression of certain genes that are required in response to acetic acid stress while general translation is compromised. To identify genes that are linked to acetic acid response by influencing this mode of translation control, we generated a manageable array of yeast gene knockout strains and subjected them to acetic acid and heat shock sensitivity analysis. This array contains 384 yeast strains, each containing a different deletion of a gene that has been linked to the process of protein synthesis. We termed this collection the translation array. We observed that deletion of either *DOM34* or *RPL36A* increased sensitivity to both acetic acid and heat shock treatments (Fig. 1). In addition, neither of these two genes was previously connected to regulation of gene expression or translation control making them interesting targets for follow up investigations.

*DOM34* encodes for a protein that dissociates inactive ribosomes attached to mRNA in the context of mRNA quality control (Passos et al., 2009). *RPL36A* encodes for large subunit of ribosomal protein. To ensure that the observed sensitivity is caused by the deletion of target genes and not the effect of some off-target mutation, *DOM34* and *RPL36A* were placed back into the corresponding gene deletion mutants. It was observed that *DOM34* and *RPL36A* were capable of reversing the increased sensitivity observed for *dom34*Δ and *rpl36a*Δ deletion mutant strains, respectively, indicating that the observed sensitivity was caused by the deletion of *DOM34* and *RPL36A*.

**Figure 2:**
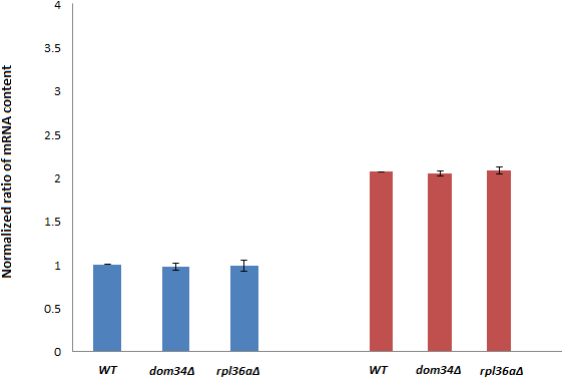
*HSP82* RNA content analyses. RT-PCR analysis was performed to study mRNA content. *HSP82* mRNA contents are related to those of control strains grown under control (blue) or exposed to acetic acid (red) conditions. *PGK1* mRNA content was used for normalization. There are no statistically significant (*P*-value ≤ 0.05) differences in mRNA contents between *WT* and tested mutants for control or acetic acid treated cells. Normalized *HSP82* mRNA contents were increased by approximately two fold in response to acetic acid exposure. The average values are obtained from at least three independent experiments.

Next, we investigated if *DOM34* and *RPL36A* can influence the expression of yeast Hsp family of proteins. *HSP82* was selected for this purpose as it has a higher induction level in comparison to *HSC82*. It is well documented that induction of *HSP82* in response to acetic acid and heat shock stress can be transcriptionally regulated (Silva et al., 2013; Borkovich et al., 1989). We examined the effect of deletion of *DOM34* and *RPL36A* on the *HSP82* transcript level. We observed that deletion of neither *DOM34* nor *RPL36A* altered the mRNA level of *HSP82* induced by acetic acid treatment (Fig. 2). These observations suggest that *DOM34* and *RPL36A* do not seem to affect the expression of *HSP82* at the mRNA level.

**Figure 3:**
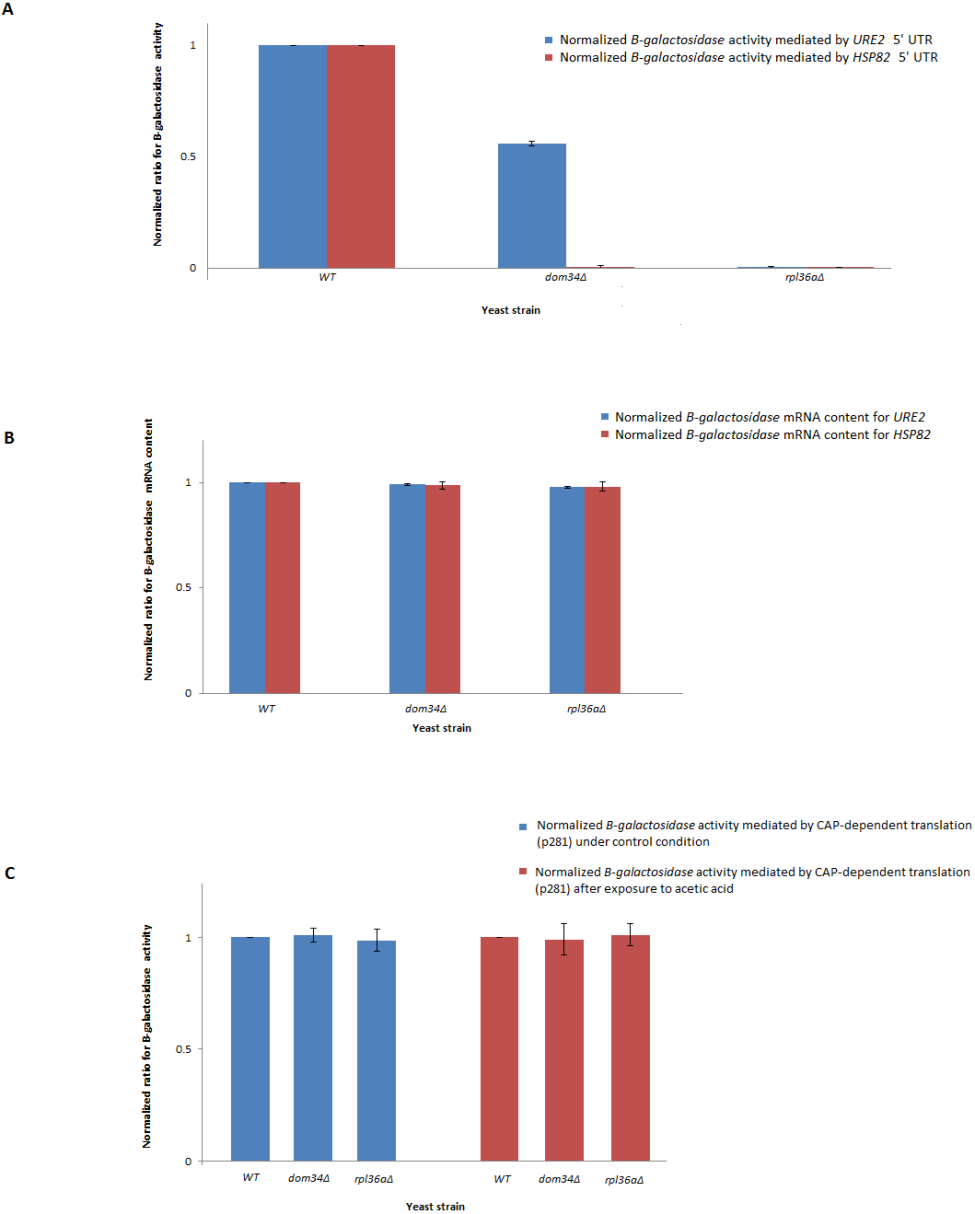
Expression analysis of *β*-*galactosidase* reporter gene. (A) Quantification of *β*-*galactosidase* expression under the control of different *HSP82* and *URE2* IRES elements. Expression levels for the mutants are normalized to the expression level of *WT* that is set to 1. Deletion of *DOM34* or *RPL36A* reduced the levels of *β*-*galactosidase* expression mediated by different IRES elements (*P*-value ≤ 0.05). (B) *β*-*galactosidase* mRNA content analysis. *β*-*galactosidase* mRNA contents are related to those of the control strain. *PGK1* mRNA content was used for normalization. There are no statistically significant differences in mRNA contents between *WT* and tested mutants. (C) CAP-dependent *β*-*galactosidase* mRNA translation. During CAP-dependent translation, when *β*-*galactosidase* mRNA translation is independent of an IRES-element, deletion of either *DOM34* or *RPL36A* has no statistically significant difference in *β*-*galactosidase* expression. The average values are obtained from at least three independent experiments.

In response to acetic acid, global translation is compromised. In contrast, the translation of selective mRNAs including *HSP82* is increased. Therefore, it remains likely that *DOM34* and *RPL36A* could influence the expression of *HSP82* at the level of translation. The selective translation of *HSP82* in response to acetic acid treatment is thought to be controlled at the level of translation initiation by a highly structured 5′ UnTranslated Region (5′-UTR) that resemble an Internal Ribosome Entry Site (IRES) structure (Silva et al., 2013). To evaluate the impact of *DOM34* and *RPL36A* on *HSP82* mRNA translation, we studied the influence of *DOM34* and *RPL36A* on translation of a quantifiable reporter gene under the translational control of *HSP82* 5′-UTR. For this, plasmid p281-4-HSP82-LacZ (Silva et al., 2013) that contains the 5′-UTR of *HSP82* in front of a *β*-*galactosidase* reporter gene which is transcriptionally controlled by an inducible GAL promoter was utilized. We observed that the acetic acid induced expression of *β*-*galactosidase* was significantly reduced when either *DOM34* or *RPL36A* was deleted (Fig. 3A). Our mRNA content analysis indicated that the observed reduction had little to do with the mRNA content as the levels of *β*-*galactosidase* mRNA were unchanged irrespective of the deletion of *DOM34* or *RPL36A* (Fig. 3B). Consequently, it appears that the expression of *β*-*galactosidase* reporter gene is affected at the translation level. As a control, when 5′-UTR of *HSP82* is removed from the expression construct (p281 construct) (Komar et al., 2003; Silva et al., 2013), the influence of *DOM34* and *RPL36A* on the expression of *β*-*galactosidase* reporter gene phased out (Fig. 3C). In this construct, *β*-*galactosidase* is translated in a CAP-dependent manner. Deletion of neither *DOM34* nor *RPL36A* affected the expression of the reporter gene which is free of the IRES-like structure.

Since *DOM34* and *RPL36A* appeared to influence translation in an *HSP82*-IRES-dependent manner, we wondered if they can influence the activity of other IRES-elements. Therefore, we investigated the role of *DOM34* and *RPL36A* in the activity of a well-characterized IRES element associated with *URE2* gene. For this, we used the p281-4-URE2-LacZ (Komar et al., 2003), expression construct containing *URE2-*IRES region in front of a quantifiable *β*-*galactosidase* reporter gene which is transcriptionally controlled by an inducible GAL promoter. Interestingly, the induced expression of *β*-*galactosidase* was also significantly reduced when either *DOM34* or *RPL36A* were deleted (Fig. 3A). As above, qRT-PCR analyses indicated that the observed alteration in LacZ expression cassette appears to be at the translational level and not at the level of mRNA content (Fig. 3B).

Altogether, these observations provide evidence that both *DOM34* and *RPL36A* seem to influence gene expression at the translation level using IRES-mediated protein synthesis. Of interest, both genes influence the expression of the investigated IRES elements; however, the level at which translation from each IRES is affected is different.

### Knockdown of PELO, human homolog of *DOM34*, reduces Hsp90 levels

**Figure 4:**
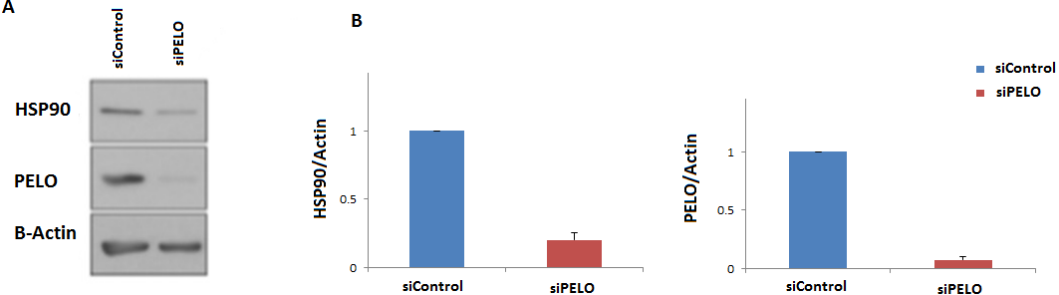
Knockdown of PELO reduced the Hsp90 levels in mammalian cells. (A) Western blot analysis of HeLa cells. (B) HeLa cells carrying non-targeting (siC) or PELO-targeting (siPELO) siRNAs indicate that when PELO is knocked down, Hsp90 levels are reduced. The average values are obtained from at least three independent experiments.

To further examine if the human *HSP90* is controlled by a similar regulatory mechanism, HeLa cells were transiently transfected with a non-targeting siRNA (siC), or an siRNA targeting the human homologue of *DOM34*, PELO (siPELO), for 48 h, and the effect of siRNA-mediated reduction of PELO on the HSP90 protein expression was assessed using western blotting (Fig. 4). We observed that reducing the levels of PELO resulted in a significant reduction of HSP90 levels. This data suggests that like in yeast, PELO regulates HSP90 expression, suggesting existence of an evolutionary conserved regulatory network.

### Genetic interaction analyses further links regulation of translation to *DOM34* and *RPL36A* activities

**Figure 5:**
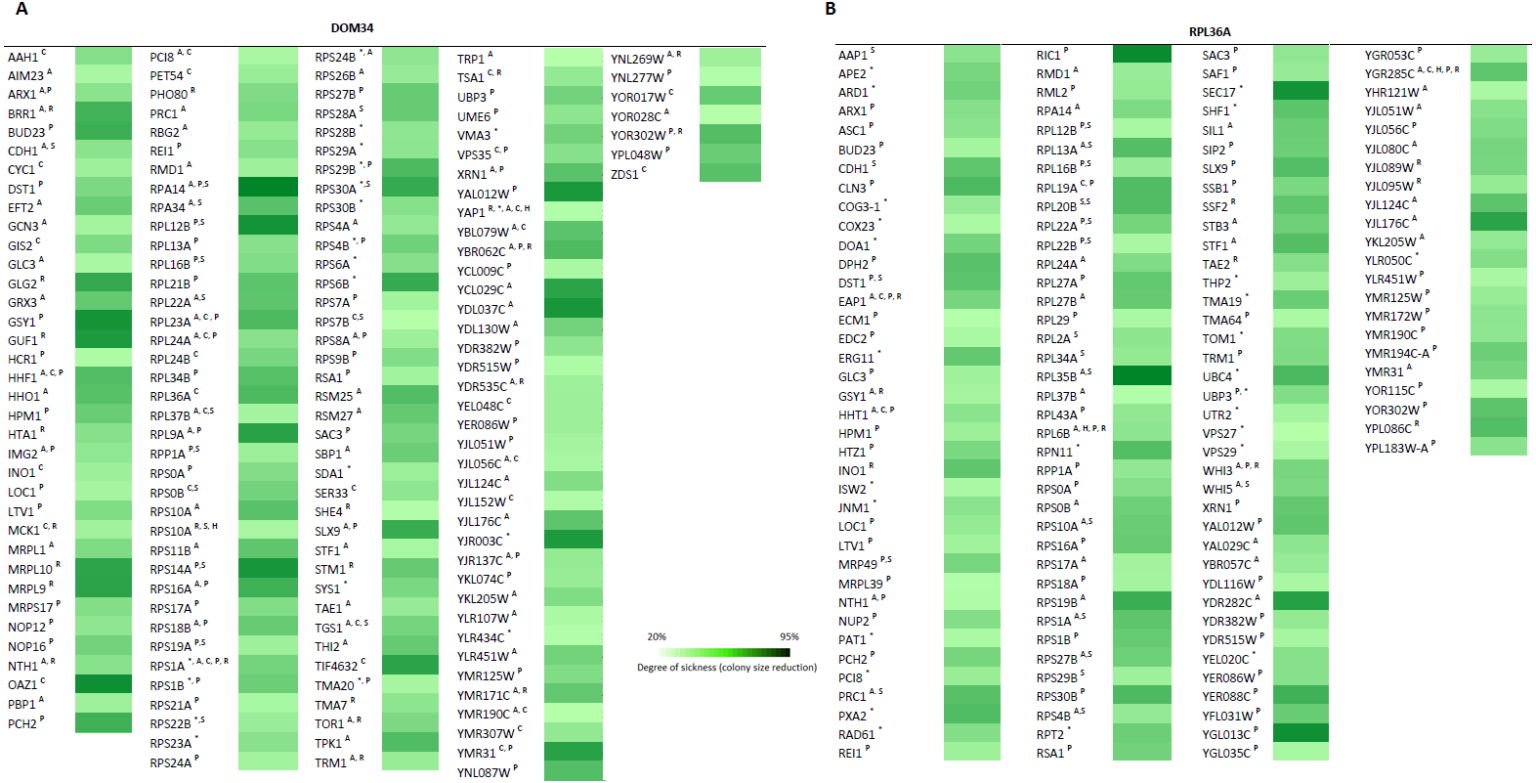
Genetic interaction analysis for *DOM34 and RPL36A.* (A) *DOM34* interacts with genes involved in ribosome biogenesis (*P*-value: 1.12E−11) (B) and *RPL36A* interacted negatively with genes linked to structural constituent of ribosome (*P*-value: 3.11E−07) Under standard laboratory conditions. Under stress conditions, new set of interactions between genes involved in translation regulation and *DOM34* (*P*-value: 3.27E−06)(A) and *RPL36A* (*P*-value: 7.47E−09)(B) are formed. ^∗^Represents interactions that were included from literature. ^A^ Represents conditional genetic interactions under acetic acid treatment (110 mM) for 2 h. ^C^ Represents conditional genetic interactions under cycloheximide (20 ng/ml) treatment. ^H^ Represents conditional genetic interactions under heat shock (37 °C) condition. ^P^ Represents conditional genetic interactions under paromomycin (10 mg/ml) condition. ^R^ Represents conditional genetic interactions under rapamycin condition (2 ng/ml). ^S^ Represents genetic interactions under standard laboratory conditions.

To further investigate the involvement of *DOM34* and *RPL36A* in regulation of translation, we studied the genetic interactions they made with genes that influence the protein synthesis pathway in yeast. Genetic interactions can be explained by alterations in expression of two genes (double mutant) that result in a phenotype, which cannot be readily justified by the phenotypes of individual gene expressions (Tong et al., 2001; Alamgir et al., 2008; Babu et al., 2001). The most commonly studied form of genetic interaction is a negative genetic interaction where double mutants have a lower growth rate (sick or lethal) than the expected individual mutant growth phenotypes. These interactions often disclose genes that are functionally related through parallel pathways (overlapping pathways). In parallel pathways, one gene/pathway can compensate the activity of the other. Consequently, deletion of both can have a significant alteration of the phenotype that is not expected from combination of the individual gene deletion phenotypes. In this context, the function of target genes may be investigated by the genetic interactions they make with other genes with known functions (Tong et al., 2001; Samanfar et al., 2013; Samanfar et al., 2014; Omidi et al., 2014). To this end, we investigated the sick phenotypes (negative interactions) that *dom34*Δ *and rpl36a*Δ formed with two sets of 384 gene deletion strains. Set one, called translation array, contains genes with known functions in different aspects of translation process and set two, called random array, that carries a variety of gene deletions (excluding those involved in translation pathway) used as a control. For this we used an approach called Synthetic Genetic Array (SGA) analysis where a targeted gene deletion strain of “*α*” mating type is crossed to a set of different gene deletion strains of the opposite mating type (“a”). After a few rounds of selection haploid double gene deletion mutants are selected (Tong et al., 2001). In this way, 768 double mutants were systematically generated for each gene in triplicate (16,128 double deletions in total). The growth fitness of double mutant gene deletion strains was quantified by colony size measurements (Samanfar et al., 2013; Samanfar et al., 2014; Memarian et al., 2007) and color-coded (Fig. 5). To have a better understanding of what these genetic interactions may imply, Gene Ontology (GO) annotation enrichment analysis on the genetic interacting partners of our target genes was performed. In this way, we evaluate the statistical enrichment of cellular function/process for the interacting partners in comparison with what could be expected by chance alone. As expected from the previously reported activity of the target genes, (*P*-value: ≤0.05). GO analysis of the interaction data indicated that *DOM34* mainly formed negative genetic interactions with genes involved in ribosomal biogenesis (*P*-value: 1.12E−11) and *RPL36A* predominantly interacted with genes involved in structural constituent of ribosome (*P*-value: 3.11E−07).

**Figure 6:**
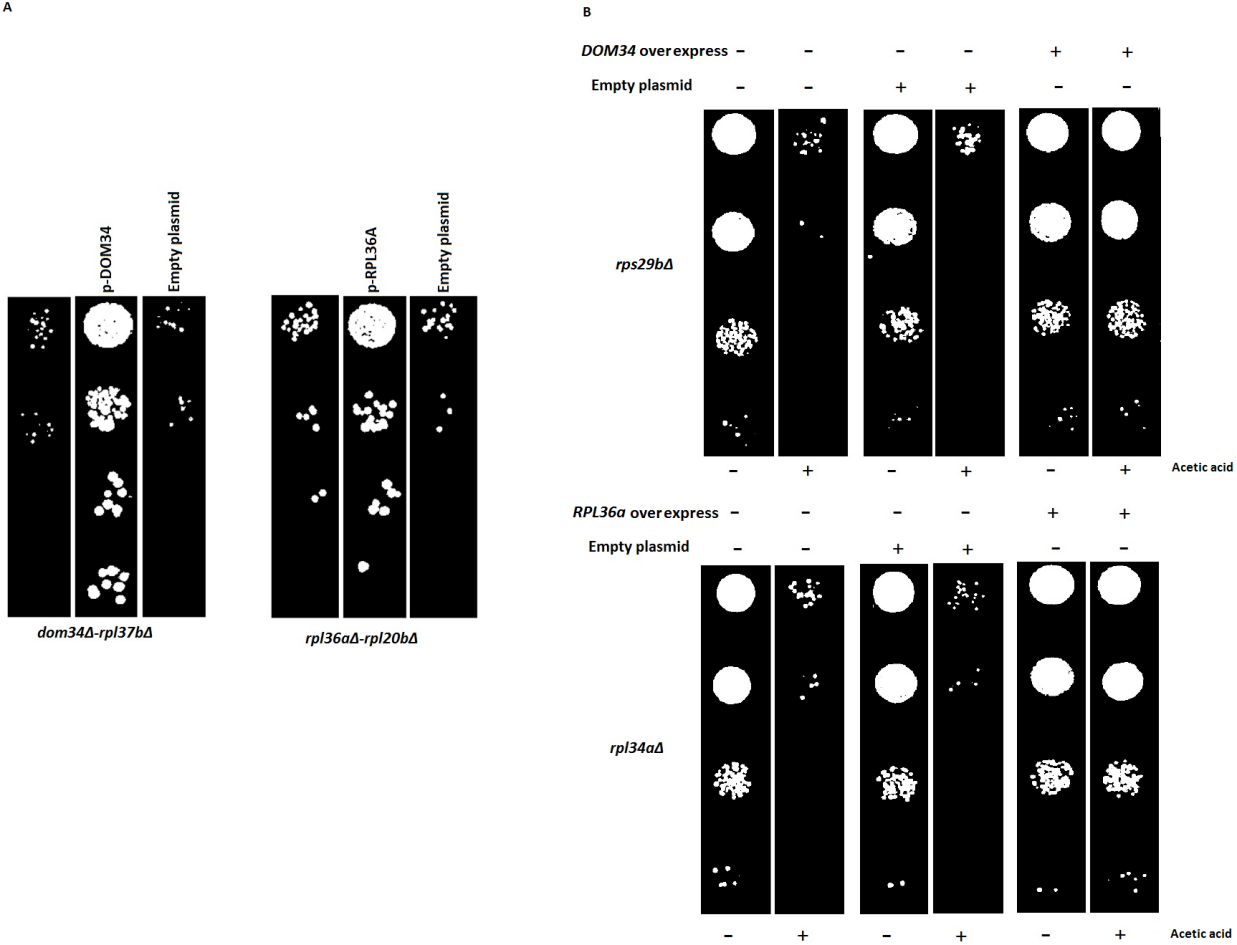
Reversal of phenotypes by re-introduction of target genes. (A) Re-introduction of deleted target genes *DOM34 and RPL36A* in double mutants. Representative examples for re-introduction of target genes in double gene deletion mutants that reverse the observed genetic interactions phenotype are shown. The sick phenotypes of double gene knockout strains are reversed when target genes are placed back into the corresponding mutant strains. (B) Spot test confirmation for phenotypic suppression analysis of *DOM34 and RPL36A.* Representative examples for gene deletion mutants (*rpl43aΔ and rps29bΔ*) with sensitivity to acetic acid (220 mM, 2 h treatment) that are compensated by overexpression of *DOM34 and RPL36A,* respectively.

Certain activities of genes may only be realized under specific physiological condition(s). These condition-dependent gene functions might be captured by genetic interactions that are specific to that condition alone (Omidi et al., 2014). In this way, stress-related function of gene X can be studied by its genetic interactions that are formed only in the presence of a particular stress. Conditional interactions are often important for the cross-communication of different pathways and can provide information about pathway regulations (Babu et al., 2001; Gagarinova et al., 2016). They highlight the mosaic nature of gene functions that vary under different physiological conditions. Such interactions would not be observed under standard laboratory growth condition (for example, the above SGA analysis). To have a better understanding of such interactions, we performed our genetic interaction analysis under targeted stress conditions including heat shock, acetic acid treatment and the presence of translation inhibitory reagents. Of interest, GO analysis of genetic interaction data under targeted stresses indicated a new additional role for both *DOM34* (*P*-value: 3.27E−06) and *RPL36A* (*P*-value: 7.47E−09) in regulation of translation. With a very high precision, these observations suggest additional roles for both *DOM34* and *RPL36A* in translation control, under stress conditions.

Since large-scale interaction analyses are prone to potential secondary mutations that might complicate the interpretation of the results, we reintroduced the target genes back into a set of double mutants. Reintroduction of *DOM34* and *RPL36A* into a representative set of corresponding double mutants (12 mutants, File S2), driven from SGA data, reversed the sick phenotype observed for the double mutants, further confirming that the observed sick phenotypes are caused by the deletion of the target genes of interest and not by a possible secondary mutation within the genome. As a pair of representative strains, our analysis with *Rpl37bΔ* and *rpl20bΔ* strains are shown in Fig. 6A (also File S2).

Next, we used phenotypic suppression array (PSA) analysis to study compensatory effect of the overexpression of the target genes (Alamgir et al., 2008). This array analysis is a similar approach to SGA with the exception that overexpression of one gene is combined with deletion of others in an array format, and that phenotypic compensation is measured in the presence of a compromising growth condition such as the presence of an inhibitory drug. We investigated the ability of the overexpression of *DOM34* and *RPL36A* genes to compensate the sick phenotype of different gene deletion strains in response to heat shock, acetic acid, cycloheximide, paromomycin and rapamycin treatments. If the overexpression of a target gene compensates the phenotype caused by the absence of another gene, a functional connection between the two genes is considered (Samanfar et al., 2014; Sopko et al., 2006; Alamgir et al., 2008; Omidi et al., 2014). To this end, the single gene deletion haploid strains in the two gene deletion array described above (translation array and random gene array) were systematically and separately transformed with overexpression plasmids for *DOM34* and *RPL36A* in addition to an empty plasmid used as a negative control. Transformed strains were grown in the presence of a sub-inhibitory concentration of acetic acid (220 mM), cycloheximide (60 ng/ml), paromomycin (22 mg/ml), rapamycin (6 ng/ml) and heat shock (37 °C). Positive hits were selected as gene deletion mutants whose sensitivity was suppressed by overexpression of *RPL36A* or *DOM34* (Fig. 6B and File S3). Of interest, we observed statistically significant enrichment of genetic interactions for both *DOM34* and *RPL36A* mainly with genes involved with ribosome biogenesis.

## Conclusion

In yeast, general translation shuts down in response to acetic acid treatment. In contrast the expression of *HSP82* and *HSC82* heat shock genes is up-regulated. This up-regulation is shown to be controlled at the level of translation and mediated by a CAP-independent manner. In the current study we identified two genes, *DOM34* and *RPL36A* that influence the *HSP82*-5′-UTR mediated translation in response to acetic acid. In addition, we show that *DOM34* and *RPL36A* can also influence the *URE2*-IRES mediated translation. Our genetic interaction analyses further support a role for these two genes in translation control in response to stress.

##  Supplemental Information

10.7717/peerj.4037/supp-1 Figure S1 Full-length uncropped blots (Figure 4)

10.7717/peerj.4037/supp-2 File S1 List of primers

10.7717/peerj.4037/supp-3 File S2 Re-introduction of deleted target genes (DOM34 and RPL36A) in double mutants Re-introduction of target genes reverses the observed genetic interactions phenotype. The sick phenotypes of double gene knockout strains are reversed when target genes are re-introduced into the corresponding mutant strains.

10.7717/peerj.4037/supp-4 File S3 List of gene deletion mutants compensated by overexpression of DOM34 and RPL36A in different stress condition Overexpression of DOM34 and RPL36A compensated for deletion of genes involved in ribosome biogenesis.

## List of Abbreviation

Apaf-1: apoptotic protease-activating factor 1
cDNA: complementary deoxy ribonucleic acid
*c-myc*: myc proto-oncogene protein
DMEM: dulbecco’s modified eagle medium
DNA: deoxy ribonucleic acid
*DOM34*: duplication of multilocus region 34
ECL: enterochromaffin-like cells
G2: Mitosis gap 2
GFP: green fluorescent protein
GST: glutathione s-transferases
GSY1: Glycogen (starch) synthase isoform 1
HRP: horseradish peroxidase
HSC82: ATP-dependent molecular chaperone heat shock chaperon 82
HSP: heat shock protein
HSP82: ATP-dependent molecular chaperone heat shock protein 82
Hsp90: heat shock protein 90
IRES: internal ribosome entry site
LacZ: lactose operon
LB: luria-bertani (Lysogeny Broth)
LiAc: lithium acetate
MATa: mating type a
MAT*α*: mating type *α*
NAT: Nourseothricin
ONPG: o-nitrophenyl-beta-D-galactopyranoside
p53: transformation-related protein 53 (TRP53)
PCD: program cell death
PCR: polymerase chain reaction
PELO: Protein pelota homolog
PGK1: 3-PhosphoGlycerate Kinase
PSA: phenotypic suppression array
PVDF: polyvinylidene difluoride
q-RT-PC: quantitative reverse real time polymerase chain reaction
RNA: ribonucleic acid
*RPL36A*: ribosomal protein large subunit 36-A
SC: synthetic complete
SDS-PAGE: sodium dodecyl sulfate-polyacrylamide gel electrophoresis
SGA: synthetic genetic array
SiRNA: small interfering RNA
YPD: yeast extract–peptone-dextrose

## Additional Information and Declarations

### Data Availability

The following information was supplied regarding data availability: The raw data has been provided as Supplemental Files.
