# Supplementary figures and images for: The sensitivity of the yeast, Saccharomyces cerevisiae, to acetic acid is influenced by DOM34 and RPL36A
Source: PeerJ. 2017 Nov 14;5:e4037. doi: 10.7717/peerj.4037 (PMC5691786; doi:10.7717/peerj.4037)

# PELO - Figure

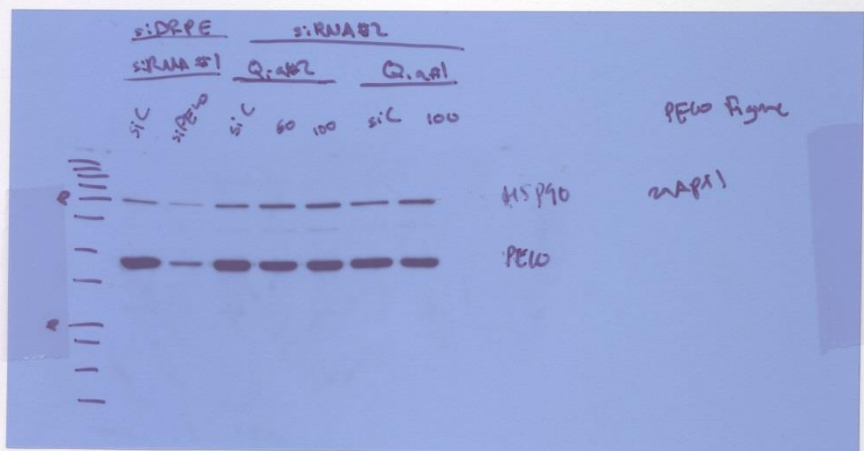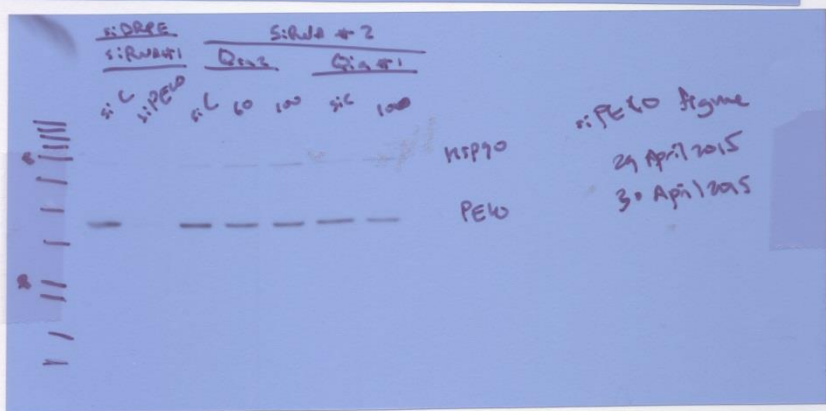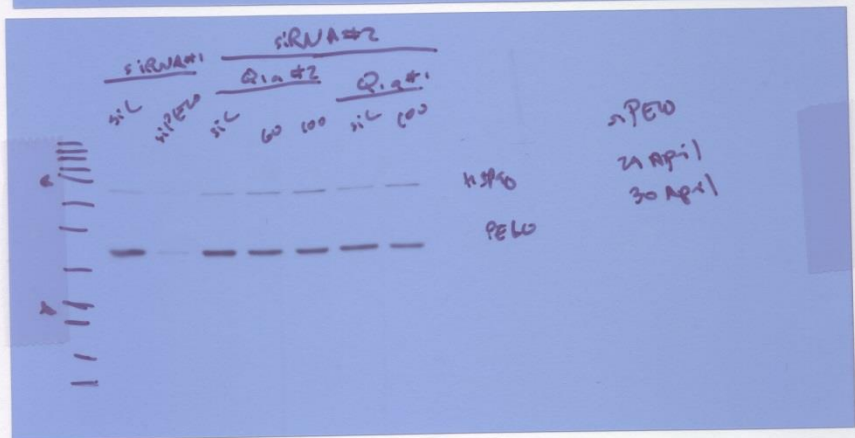

# PEW- Figure

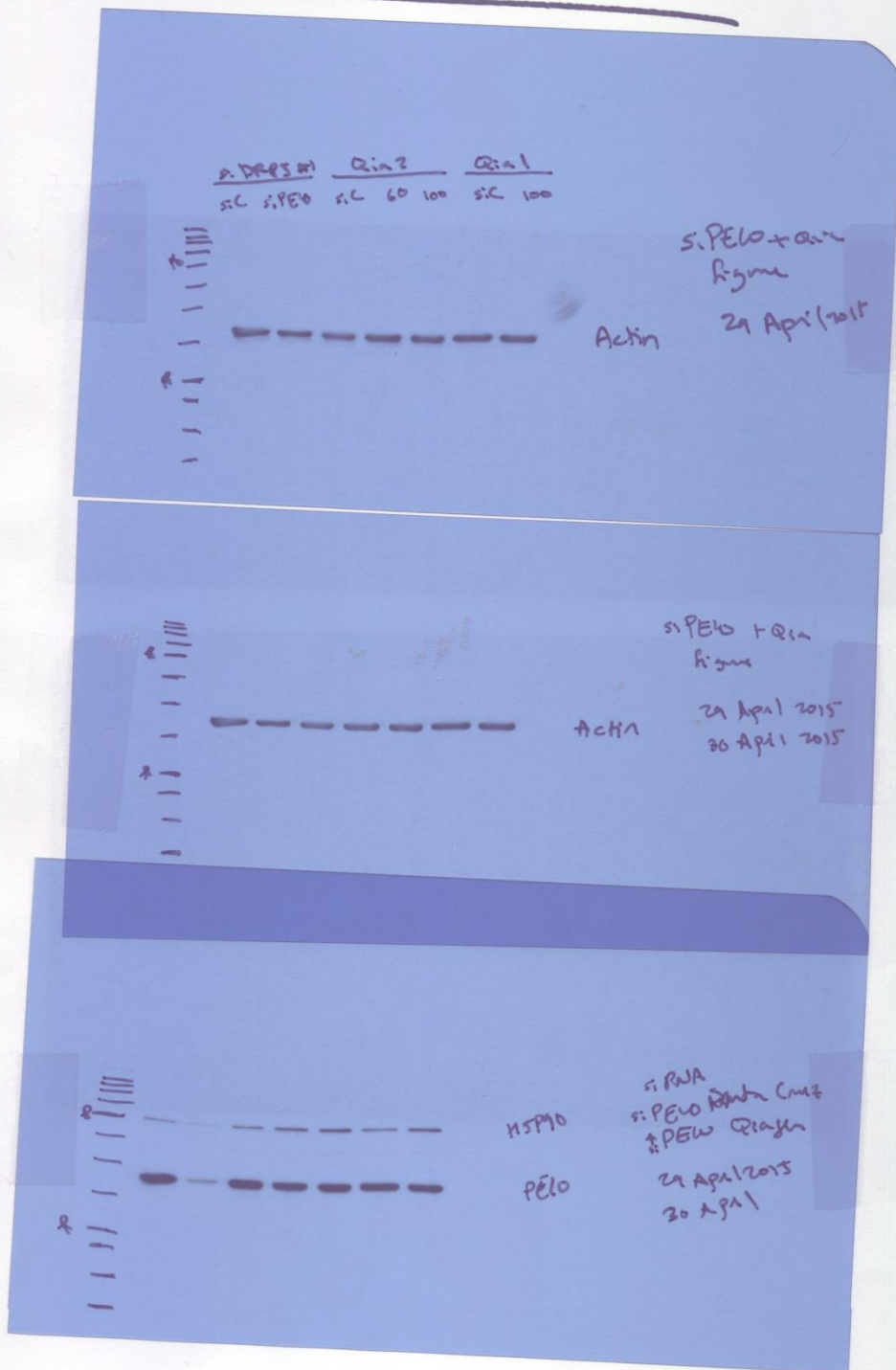

PEM - 2010

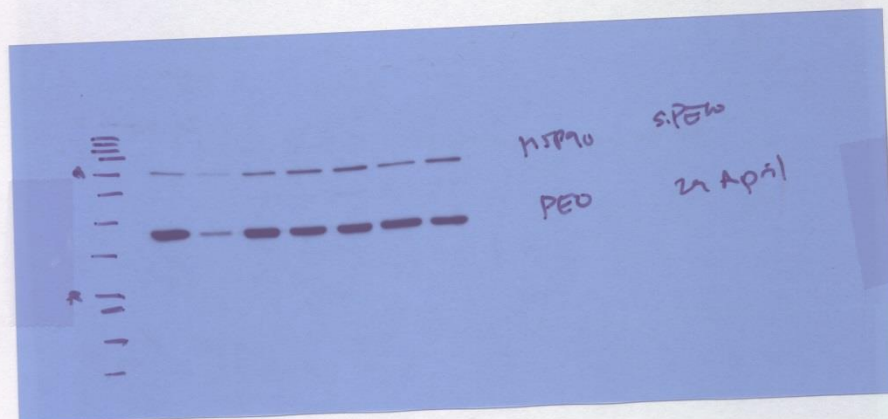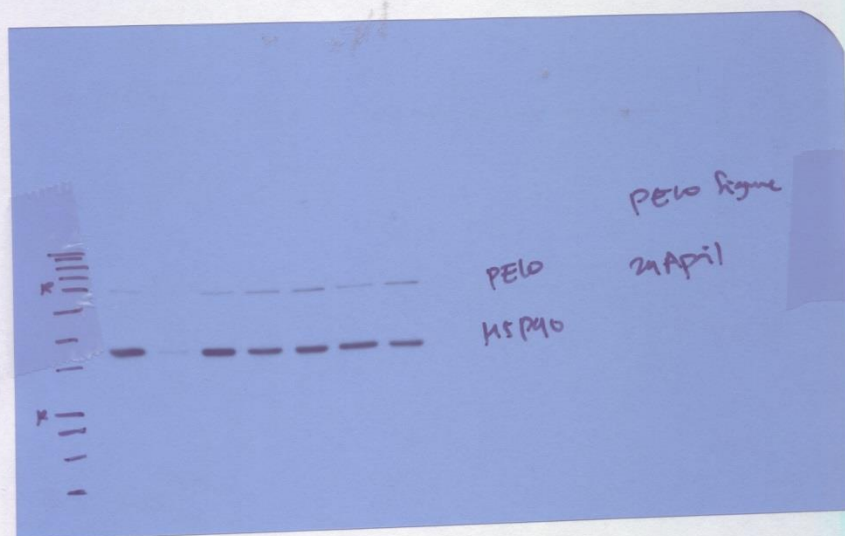

Supplement: Figure S1 [file peerj-05-4037-s001.pdf]
